# Angioplasty and/or stenting after thrombectomy in patients with large vessel occlusion associated with underlying intracranial atherosclerotic stenosis: a meta-analysis and systematic review

**DOI:** 10.1007/s00701-025-06690-6

**Published:** 2025-10-16

**Authors:** Hesham Kelani, Hazem Mohamed Salamah, Eli Berglas, Emina Dzafic, Shivasuryan Vummidi, Huzaifa Dorria, Emily Wen Jing Shuai, Gitanjali Reddy, Desen Zeng, Ariel Makower, Dylan Davie, Amber Khemlani, Diana Greene-Chandos, Volodymyr Vulkanov, David Rosenbaum-HaLevi, David P. Lerner, Lisa R. Merlin, Priyank Khandelwal

**Affiliations:** 1 Department of Neurology, SUNY Downstate Health Sciences University at One Brooklyn Health, Brooklyn, NY USA; 2 Faculty of Medicine, Zagazig University, Zagazig, Egypt; 3 Touro College of Osteopathic Medicine, Touro University, New York, NY USA; 4 School of Medicine, SUNY Downstate Health Sciences University, Brooklyn, NY USA; 5 Department of Neurology, School of Medicine, University of Saint Louis, St. Louis, MO USA; 6 Department of Neurology, Rutgers, New Jersey School of Medicine, Newark, NJ USA; 7 Departments of Neurology, Pharmacology and Physiology, SUNY Downstate Health Sciences University, Brooklyn, NY USA; 8 Department of Neurosurgery, Rutgers, New Jersey School of Medicine, Newark, NJ USA

**Keywords:** Angioplasty, Stent, Mechanical thrombectomy, Atherosclerosis, Large vessel

## Abstract

**Background:**

Intracranial atherosclerotic stenosis-related large vessel occlusion (ICAS-LVO) is a leading cause of acute large vessel occlusion stroke. For patients suffering from ICAS-LVO stroke, the first-line treatment is mechanical thrombectomy (MT). However, whether balloon angioplasty and/or stenting should be performed after MT in patients with ICAS-LVO stroke remains uncertain in clinical practice. In this study, a meta-analysis and systematic review was performed to assess the efficacy and safety of angioplasty and/or stenting after MT.

**Methods:**

A thorough search of the PubMed, Web of Science, Cochrane, and Scopus databases from inception to February 28, 2025, was performed to retrieve clinical trials comparing angioplasty and/or stenting plus MT versus MT alone. Vessel recanalization, functional independence at 90 days, early neurological deterioration, symptomatic intracranial hemorrhage (ICH), asymptomatic ICH, any ICH, and mortality at 90 days were analyzed. RevMan version 5.4 was used to calculate the pooled risk ratio (RR) and 95% confidence interval (CI).

**Results:**

The analysis included nine studies. No significant difference was observed between MT + angioplasty/stenting and MT alone in terms of vessel recanalization (RR = 1.03, 95%CI = 0.97–1.11, p = 0.33), symptomatic ICH (RR = 1.06, 95%CI = 0.65–1.71, *p* = 0.82), asymptomatic ICH (RR = 1.17, 95%CI = 0.86–1.60, *p* = 0.33), any ICH (RR = 0.97, 95%CI = 0.77–1.21, *p* = 0.79), and mortality at 90 days (RR = 0.97, 95%CI = 0.70–1.34, p = 0.84). Angioplasty and/or stent plus MT significantly increased the likelihood of functional independence (mRS 0–2) at 90 days (RR = 1.24, 95%CI = 1.11–1.38, *p* < 0.001) and decreased early neurological deterioration (RR = 0.60, 95% CI = 0.37–0.95, *p* = 0.03).

**Conclusion:**

Angioplasty and/or stent after MT was associated with significantly better functional independence and reduced early neurological deterioration compared to MT alone a without significant adverse events.

**Supplementary Information:**

The online version contains supplementary material available at 10.1007/s00701-025-06690-6.

## Introduction

Ischemic stroke contributes to considerable morbidity and mortality worldwide. In 2021, ischemic stroke had an estimated 92.4 incident cases and 44.2 deaths per 100,000 persons [15]. Despite the high disease burden of ischemic stroke, the incidence is expected to continue rising [22]. Approximately 20% of ischemic strokes are due to large vessel occlusion (LVO) [24]. Ischemic strokes due to LVO lead to greater morbidity and mortality than ischemic strokes without LVO [18]. Intracranial atherosclerotic stenosis (ICAS) is a leading cause of LVO stroke [2, 11]. The rates of intracranial atherosclerotic stenosis-related large vessel occlusion (ICAS-LVO) vary greatly. The variation in ICAS-LVO rates across studies is primarily due to disparities in the racial makeup and underlying vascular risk factors of ICAS present in various patient populations [7]. Therefore, a cumulative analysis of several patient populations would provide more generalizable findings. One such area that requires more study in ICAS-LVO is the primary treatment.

The primary treatment of ICAS-LVO is mechanical thrombectomy (MT) [14]. While MT is considered the gold-standard treatment option for ICAS-LVO, the underlying ICAS presents distinct challenges. As one meta-analysis found, the odds of reocclusion in ICAS-LVO are more than 20-fold greater than in patients without ICAS [23]. Additionally, the presence of ICAS increases the risk of recurrent stroke [11]. Given the high rates of reocclusion, the efficacy of MT with angioplasty and/or stenting (MT + angioplasty/stent) has been explored. However, there is limited clinical trial data on the efficacy of MT + angioplasty/stent [7]. Due to the high rates of reocclusion, morbidity, and mortality of ICAS-LVO, there is a need to explore vessel-directed treatment modalities such as using angioplasty and/or stenting. Furthermore, the highly variable population-dependent nature of ICAS necessitates a multi-population approach. Therefore, the current study conducted a systematic review and meta-analysis to determine the efficacy and safety of MT + angioplasty/stent versus MT alone in patients with ICAS-LVO.

## Methods

The study was reported using the PRISMA guidelines for reporting systematic reviews and meta-analyses of randomized controlled trials (RCTs) [20]. We prospectively registered our study in the International Prospective Register of Systematic Reviews (PROSPERO), registration identifier CRD420251010571, https://www.crd.york.ac.uk/PROSPERO/view/CRD420251010571.

### Eligibility criteria

The following criteria were used to determine which studies were eligible: patients, intervention, control, and outcomes (PICO). The participants in this study were patients with large vessel occlusion due to underlying intracranial atherosclerosis (ICAS) and underwent mechanical thrombectomy. Angioplasty and/or stent after mechanical thrombectomy was the intervention of interest, and the comparator was mechanical thrombectomy alone without angioplasty or stent. In order to be included in the study, the studies must have measured and reported the outcomes of interest. Due to the limited number of available studies, both randomized controlled trials (RCTs) and comparative observational studies were accepted. No restrictions were placed on the race, country, or publication time of the studies. Additionally, no limits were placed on the follow-up time. Studies involving animals, non-English language papers, reviews, and single-arm studies were excluded. Patients with large vessel occlusion without underlying atherosclerosis (due to causes other than atherosclerosis, such as dissection) were excluded.

### Information sources

A thorough search of the PubMed, Web of Science, Cochrane, and Scopus databases from inception to February 28, 2025, revealed relevant publications. The reference lists of the included articles were reviewed to determine any additional relevant studies.

### Search strategy

A combination of the following terms was used in the database search: (Angioplasty OR stent*) AND (thrombectomy OR embolectomy) AND (atherosclerotic OR atherosclerosis). No filters were applied.

### Selection process

All records were assembled using Endnote and then moved to an Excel file, where a two-step process to retrieve eligible studies was performed. The first step was title and abstract screening; articles passed this step and moved on to the full-text screening phase. The viability of each article in each phase was independently evaluated by two authors before the findings were discussed. A third senior author resolved any disagreements.

### Data collection process

The lead author created formatted Excel sheets containing baseline information, study characteristics, and outcomes of interest. Two authors separately extracted and discussed the data from each study. A third senior author resolved any disagreements that arose.

### Data items (outcomes)

The primary outcomes were vessel recanalization and functional independence at 90 days (mRS = 0–2 and mRS = 0–1). The secondary outcomes were early neurological deterioration, symptomatic intracranial hemorrhage (ICH), asymptomatic ICH, any ICH, and mortality at 90 days.

### Data items

Two review authors extracted the characteristics of the studies and baseline data, which included study ID, country (center), study design, patient characteristics, degree of stenosis, site of vessel occlusion, sample size, age, gender, baseline comorbidities.

### Study risk of bias assessment

The Newcastle–Ottawa Scale (NOS) [17] was used to evaluate the quality of the observational studies. The overall scores assigned to the studies, ranging from 0 to 10 points, determined their quality, with very good quality studies receiving a score of 9–10 points, good quality studies receiving a score of 7–8 points, satisfactory quality studies receiving a score of 5–6 points, and unsatisfactory quality studies receiving a score of 0–4 points. Two authors independently evaluated the quality of each study prior to conducting the discussion, and any disagreements were resolved by a third senior author.

### Statistical analysis

We conducted a pairwise meta-analysis using RevMan version 5.4. All data were collected as event and total. We calculated the pooled risk ratio (RR) and 95% confidence interval (CI). Statistical heterogeneity was assessed using I^2^ and the chi-squared test. The heterogeneity was considered significant if *p* < 0.05 or I^2^ > 60%. We used a random effect model if there was significant heterogeneity; otherwise, we used the fixed effect model. We excluded one study from any analyses with high heterogeneity and re-evaluated the pooled estimate and heterogeneity. Subgroup analysis was performed based on the degree of stenosis and type on intervention. Degree of stenosis in the included studies varied: > 50%, > 70%, and > 90%. However, subgroup of those with > 70% stenosis was the only subgroup that could be analyzed as the other subgroups had only one study. Type of intervention varied among studies. Some studies used stent in all of their patients with or without angioplasty, other studies didn’t provide separate data for those with stent and/or angioplasty. We performed subgroup of studies used stent in all of their patients with or without angioplasty.

Publication bias was examined visually through inspecting funnel plot, with symmetrical funnel plot indicating no publication bias. Egger tests were conducted using R to quantitatively assess publication bias, where significant p values (*p* < 0.05) indicate publication bias.

## Results

### Literature search

**Fig. 1:**
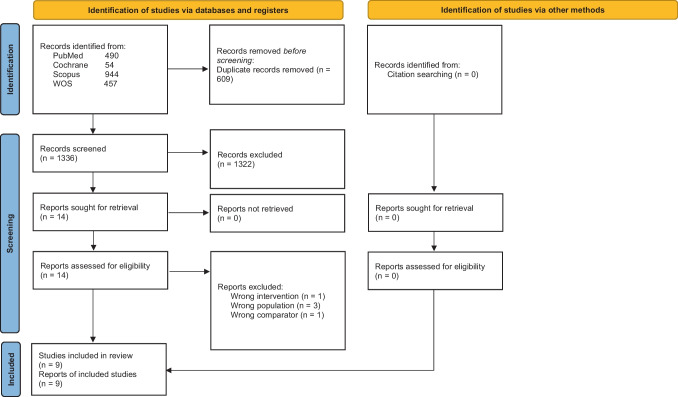
PRISMA flow diagram

Our systematic search across multiple databases yielded a total of 1945 studies. After removing 609 duplicates, we proceeded to screen the remaining 1336 records based on their titles and abstracts. This initial screening process resulted in the exclusion of 1322 studies that did not meet our inclusion criteria. Consequently, 14 studies were deemed eligible for full-text evaluation. Five studies were excluded following a comprehensive review of their full texts, leaving nine studies [1, 3, 4, 8, 10, 12, 16, 25, 27] that met the inclusion criteria and were included in our meta-analysis. The PRISMA flow diagram records search and selection are outlined in Fig. 1.

### Study characteristics

Table 1 summarizes the key characteristics of the included studies, which were conducted across multiple countries, including China, France, and the United States, using both prospective and retrospective cohort designs. The studies focused on patients with large vessel occlusion (LVO) related to intracranial atherosclerotic stenosis (ICAS) who underwent mechanical thrombectomy (MT), with or without additional angioplasty and/or stenting. The degree of stenosis varied across studies, with most defining significant stenosis as ≥ 70%. The site of vessel occlusion primarily involved the intracranial internal carotid artery (ICA) and middle cerebral artery (MCA), though some studies also included posterior circulation occlusions affecting the vertebral and basilar arteries (Table 2).

**Table 1:** Summary of the included studies

Study ID	Country	Study design	Patient characteristics	Degree of stenosis	Site of vessel occlusion	Type of intervention
Wu et al. 2019 [25]	China (Multicenter)	Prospective Cohort Study	Patients with ELVO in anterior cerebral circulation due to ICAS > 70% that underwent MT	stenosis > 70%	ICA (24.8%), MCA M1 (75.2%)	Angioplasty only in 31% of cases, stent alone in 6%, balloon-mounted stent in 63%
Zhang et al. 2022 [27]	China (Multicenter)	Prospective Cohort Study	Patients with ICAS-LVO that received endovascular treatment	stenosis > 70% or stenosis > 50% with impaired distal blood flow	Anterior Circulation (66.9%), Posterior Circulation (33.1%))	Angioplasty and/or permanent stenting. Stents were balloon-mounted stenting, or self-expanding stent
Abdelrady et al. 2023 [1]	France (Multicenter)	Retrospective Cohort Study	Caucasian ICARO patients that were treated with endovascular therapy	> 70% narrowing or stenosis of any degree with propensity for reocclusion	Intracranial ICA (17%), MCA (46%), ICVA-BA (37%)	Angioplasty only in 40.5% of cases, self-expanding stent in 18%, balloon-mounted stent in 41.5%
Li et al. 2023 [16]	China (Single Center)	Retrospective Cohort	Patients 18–80 with LVO related to underlying > 70% ICAS following mechanical thrombectomy ± stenting/angioplasty	Stenosis ≥ 70%	ICA (38.6%) MCA (61.4%) Anterior circulation (100%)	About 59% underwent only balloon angioplasty, and 41% cases in addition needed stenting with balloon-mounted stenting or self-expanding stent
Hassan et al. 2022 [12]	United States (Single Center)	Retrospective Cohort	Patients with LVO with underlying ICAD who had undergone mechanical thrombectomy ± stenting	Stenosis > 50%	ICA (10.7%) MCA M1/M2 (52.1%) ICA + MCA (26.7%) PCA (2.6%) Basilar (7.9%) Anterior circulation (89.5%) Posterior circulation (10.5%)	All received stent. Angioplasty using the Gateway PTA balloon catheter was done in 82.6% of cases
Anadani et al. 2021 [3]	France (ETIS registry) (Multicenter) & international (TITAN Registry) (Multicenter)	Retrospective Cohort	Patients 18 or older with anterior circulation tandem occlusions, caused by either atherosclerosis or dissection, involving cervical ICA lesion and proximal intracranial lesion (ICA/MCA), treated with mechanical thrombectomy ± cervical ICA stenting/angioplasty	Stenosis ≥ 90%	Anterior circulation (100%)	Intracranial rescue stenting with or without balloon angioplasty
Deng et al. 2023 [8]	China (Multicenter)	Prospective Cohort Study	Patients with intracranial atherosclerotic disease related large-vessel occlusion strokes	Stenosis > 70%	ICA (20.4%) MCA (47.5%) vertebral artery (30.3%)	Balloon angioplasty alone, balloon-mounted stenting, or self-expanding stent
Guo et. Al. 2023 [10]	China (Single Center)	Retrospective study	Patients received endovascular treatment in Zhejiang Provincial People’s Hospital. Patients were 18 and older, had an onset-to-puncture time ≤ 24 h, has a mRS of < 2 before stroke, and had AIS caused by ICAS-LVO	Stenosis > 70%	ICA (27.6%) MCA (51.2%) basilar artery (9.4%) vertebral artery (11.8%)	All received stent. If necessary, high-pressure balloon angioplasty was performed before and after stent placement
Baek et al. 2021 [4]	China (Multicenter)	Retrospective study	Patients 18 years old or older with an intracranial LVO of anterior circulation who have had consecutive acute strokes, and whose first endovascular treatment was mechanical thrombectomy	Stenosis > 70%	ICA (24.5%) MCA (75.5%)	Intracranial rescue stenting with or without balloon angioplasty

**Table 2:** Baseline characteristics

Study ID	Arms	Sample size	Age Mean ± SD	Women *N* (%)	Diabetes mellitus *N* (%)	Hypertension *N* (%)	History of stroke *N* (%)	Smoking *N* (%)	IV thrombolysis *N* (%)	NIHSS at admission Mean ± SD	General anesthesia *N* (%)
Wu et al. 2019 [25]	MT with angioplasty and/or stenting	81	63.1 ± 10.4	27 (33.3%)	21 (25.9%)	49 (60.4%)	15 (18.5%)	26 (32.1%)	28 (34.5%)	19.3 ± 6.0	57 (70.4%)
	MT alone	32	65.2 ± 12.9	13 (40.6%)	11 (34.3%)	21 (65.6%)	7 (21.9%)	8 (25.0%)	10 (31.2%)	16.3 ± 6.2	16 (50.0%)
Zhang et al. 2022 [27]	MT with angioplasty and/or stenting	182	59.7 ± 11.2	31 (17%)	31 (17.0%)	125 (68.7%)	44 (24.2%)	84 (46.2%)	41 (22.5%)	16 ± 9.0	76 (41.8%)
	MT alone	175	64 ± 14.2	47 (26.9%)	38 (21.7%)	106 (60.6%)	33 (18.9%)	72 (41.1%)	29 (16.6%)	17.7 ± 7.5	70 (40.0%)
Abdelrady et al. 2023 [1]	MT with angioplasty and/or stenting	94	70.3 ± 15.0	34 (36.2%)	NA	53 (56.4%)	11 (11.7%)	23 (24.5%)	23 (24.5%)	15.3 ± 7.7	55 (58.5%)
	MT alone	36	71.3 ± 10.0	16 (44.4%)	NA	24 (67%)	4 (11%)	15 (42%)	10 (28%)	13.3 ± 8.5	16 (44%)
Li et al. 2023 [16]	Angioplasty + MT	64	61.1 ± 10.3	18 (28.1)	14 (21.9)	45 (70.3)	NA	30 (46.9)	29 (45.3)	15 ± 4.55	31 (48.4)
	MT alone	120	62.5 ± 12.3	37 (30.8)	25 (20.8)	80 (66.7)	NA	50 (41.7)	46 (38.3)	15.17 ± 6.00	43 (35.8)
Hassan et al. 2022 [12]	Stenting + MT	46	70.34 ± 13.75	17 (37)	28 (60.9)	40 (87)	10 (21.7)	5 (10.9)	7 (15.2)	16.19 ± 7.97	NA
	MT Alone	374	70.64 ± 12.92	180 (48.1)	168 (44.9)	333 (89)	91 (24.3)	35 (9.4)	152 (40.6)	16.35 ± 8.12	NA
Anadani et al. 2021 [3]	MT + Stenting	341	63.4 ± 12.9	115 (33.7)	44 (13.0)	195 (57.1)	NA	113 (33.2)	214 (62.9)	15.67 ± 6.70	176 (51.7)
	MT Alone	262	62.3 ± 13.7	80 (30.5)	40 (15.4)	127 (48.5)	NA	92 (35.2)	166 (63.4)	15.67 ± 5.22	79 (30.2)
Deng et al. 2023 [8]	Late Angioplasty and/or stenting	93	60 ± 10.37	75 (80.6)	22 (23.8)	59 (62.9)	NA	38 (41.1)	23 (24.4)	15	15 (16.6)
	MT Alone	178	61 ± 12.59	149.55 (79.7)	42 (22.6)	120 (63.8)	NA	97 (51.8)	36 (19.1)	16	33 (17.5)
Guo et. Al. 2023 [10]	MT + Stenting	41	61.98 ± 13.70	10 (24.39)	10 (24.4%)	29 (70.7%)	NA	13 (31.7%)	18 (43.9%)	15	39 (95.1%)
	MT Alone	86	63.16 ± 13.39	23 (26.74)	22 (25.6%)	64 (74.4%)	NA	29 (33.7%)	29 (34.1%)	16	76 (88.4%)
Baek et al. 2021 [4]	MT + Stenting	25	69.7 (± 15.6)	9 (36)	8 (32.0)	14 (56.0)	25 (100)	6 (24.0)	1 (4.0)	17	0
	MT Alone	24	74.6 (± 11.9)	13 (54.2)	8 (33.3)	18 (75.0)	24 (100)	5 (20.8)	0 (0.0)	15.5	0

### Risk of bias assessment

Quality of the included studies ranged from good quality (8 scores) in four studies [1, 10, 25, 27] and very good quality (9 scores) in five studies [3, 4, 8, 12, 16]. Table 3 shows the quality assessment of the included studies.

**Table 3:** Risk of bias assessment

Study ID	Selection				Comparability	Outcome			Total Score
	Representativeness of the exposed cohort	Selection of the non-exposed cohort	Ascertainment of exposure	Demonstration that outcome of interest was not present at start of study	Comparability of cohorts on the basis of the design or analysis	Assessment of outcome	Was follow-up long enough for outcomes to occur	Adequacy of follow up of cohorts	
Wu et al. 2019 [25]	*	*	*	*	*	*	*	*	8
Zhang et al. 2022 [27]	-	*	*	*	**	*	*	*	8
Abdelrady et al. 2023 [1]	*	*	*	*	*	*	*	*	8
Li et al. 2023 [16]	*	*	*	*	**	*	*	*	9
Hassan et al. 2022 [12]	*	*	*	*	**	*	*	*	9
Anadani et al. 2021 [3]	*	*	*	*	**	*	*	*	9
Deng et al. 2023 [8]	*	*	*	*	**	*	*	*	9
Guo et. Al. 2023 [10]	-	*	*	*	**	*	*	*	8
Baek et al. 2021 [4]	*	*	*	*	**	*	*	*	9

## Outcomes

### Vessel recanalization

**Fig. 2:**
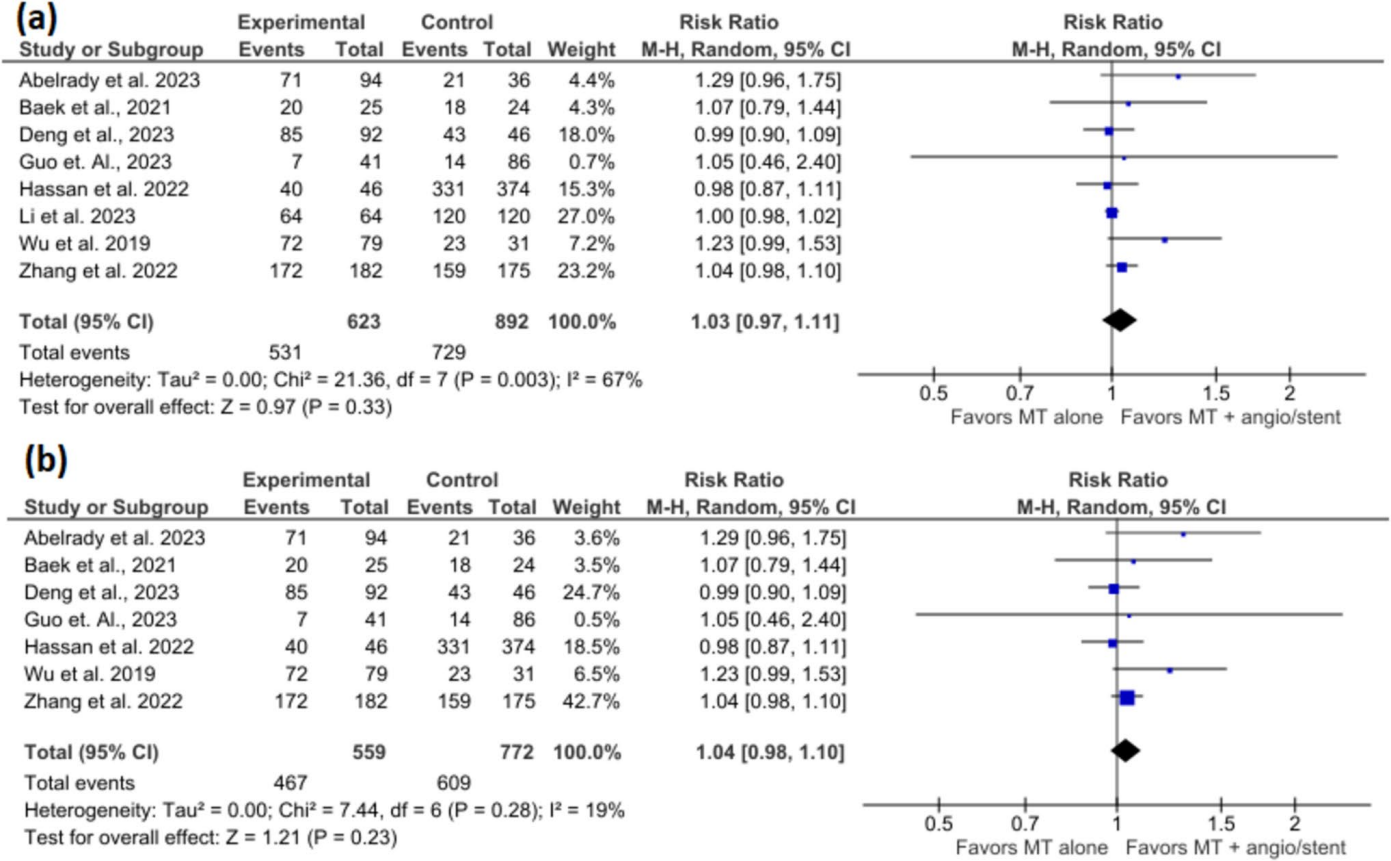
Forest plots show (**a**) vessel revascularization and (**b**) sensitivity analysis after excluding Li et al. 2023

The meta-analysis included 8 studies with 623 participants in the MT + angioplasty/stent group and 892 participants in the MT alone group. The pooled estimate showed no significant effect of MT + angioplasty/stenting in terms of vessel recanalization compared to MT alone (RR = 1.03, 95% CI = 0.97–1.11, *p* = 0.33) (Fig. 2a). There was a significantly high heterogeneity (I^2^ = 67%, *p* = 0.003).

**Fig. 3:**
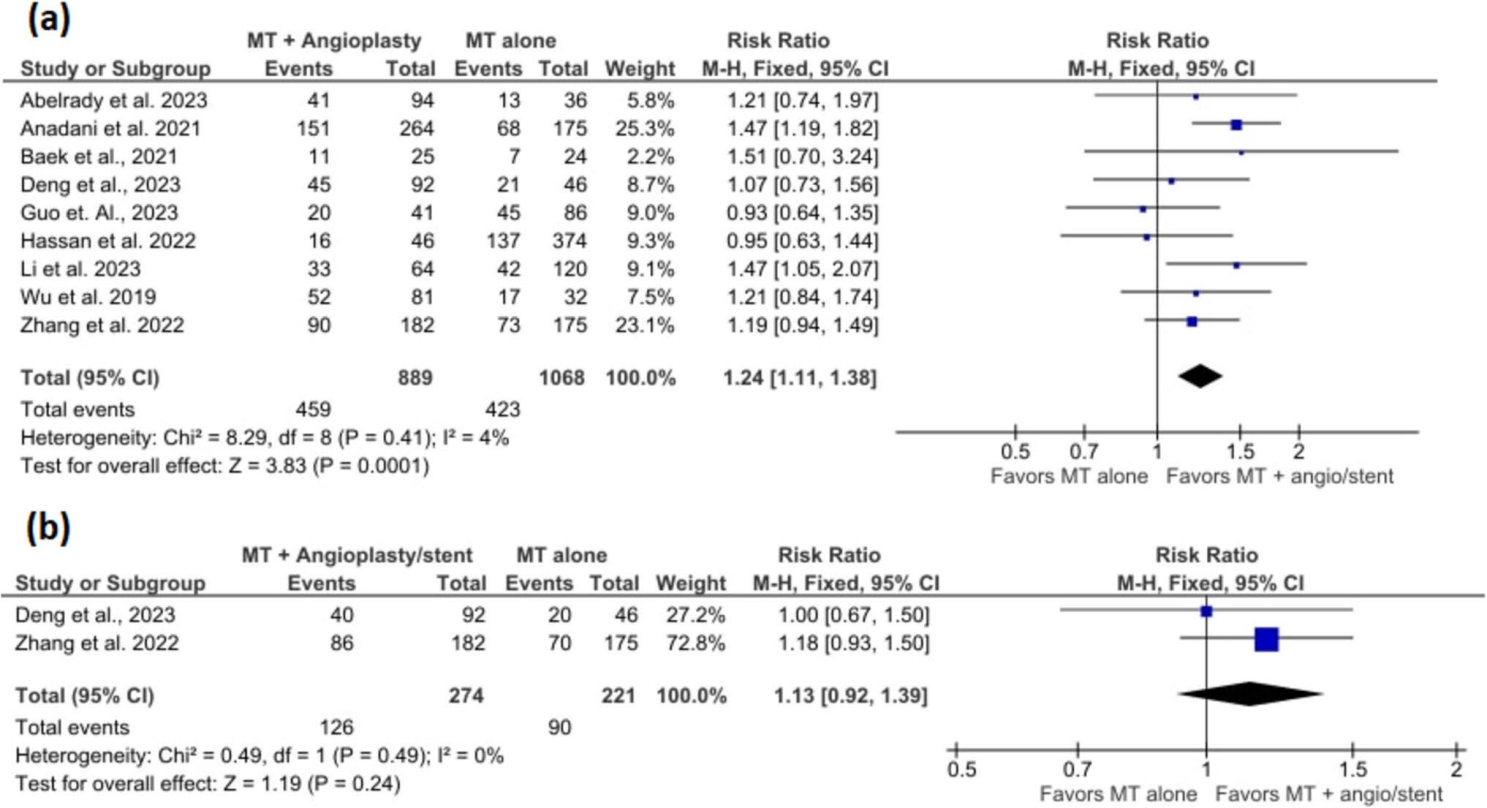
Forest plots show (**a**) functional Independence at 90 days (mRS 0–2) and (**b**) functional Independence at 90 days (mRS 0–1)

After excluding Li et al. 2023 [16], the pooled estimate continued to show no significant difference between both groups (RR = 1.04, 95 CI = 0.98–1.10, *p* = 0.28), but the heterogeneity was reduced to 19% (I^2^ = 19%, *p* = 0.28) (Fig. 3b).

### Functional independence at 90 days (mRS 0–2)

The meta-analysis included 9 studies with 889 participants in the MT + angioplasty/stent group and 1068 participants in the MT alone group. The pooled estimate showed that MT + angioplasty/stent was associated with a 1.24-times increased likelihood of functional independence at 90 days (mRS = 0–2) compared to patients who only received MT (RR = 1.24, 95% CI = 1.11–1.38, *p* < 0.001) (Fig. 3a). There was no significantly high heterogeneity (I^2^ = 4%, *p* = 0.41).

### Functional independence at 90 days (mRS 0–1)

The meta-analysis included 2 studies with 274 participants in the MT + angioplasty/stent group and 221 participants in the MT alone group. The pooled estimate showed no significant effect of MT + angioplasty/stenting and functional independence at 90 days (mRS = 0–1) compared to patients with MT alone (RR = 1.13, 95% CI = 0.92–1.39, *p* = 0.24) (Fig. 3b). There was no significantly high heterogeneity (I^2^ = 0%, *p* = 0.49).

### Early neurologic deterioration

**Fig. 4:**
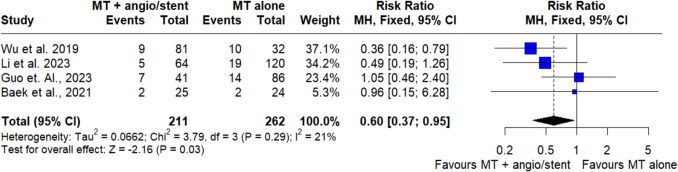
Forest plot shows early neurological deterioration

The meta-analysis included 4 studies with 211 participants in the MT + angioplasty/stent group and 262 participants in the MT alone group. The pooled estimate showed that angioplasty/stenting significantly reduced the risk of early neurological deterioration (RR = 0.60, 95% CI = 0.37–0.95, *p* = 0.03) (Fig. 4). There was no significantly high heterogeneity (I^2^ = 21%, *p* = 0.29).

### Symptomatic ICH

**Fig. 5:**
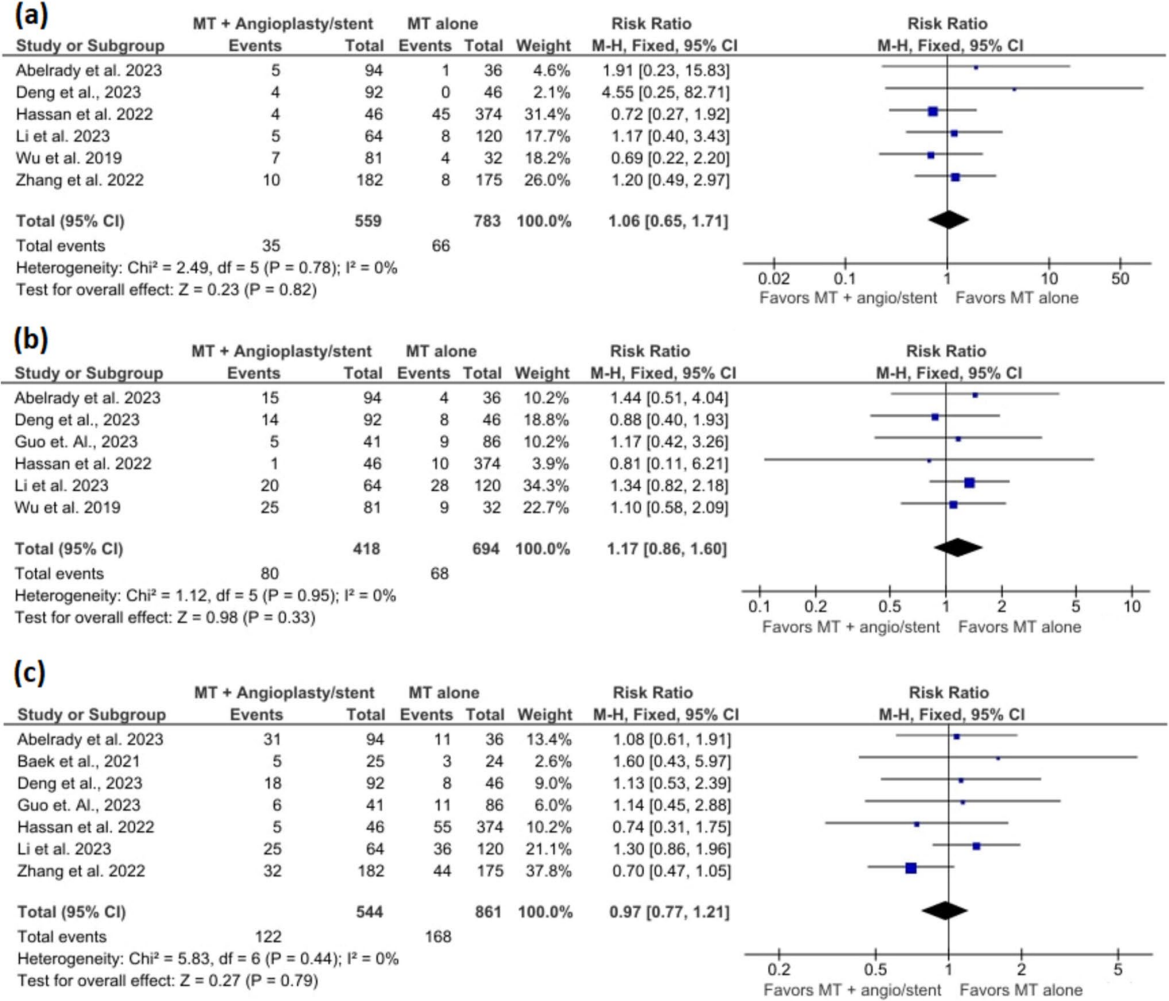
Forest plots show (**a**) symptomatic ICH, **b** asymptomatic ICH, and (**c**) any ICH

The meta-analysis included 6 studies with 559 participants in the MT + angioplasty/stent group and 783 participants in the MT alone group. The pooled estimate showed no significant association of angioplasty/stenting and symptomatic ICH (RR = 1.06, 95% CI = 0.65–1.71, *p* = 0.82) (Fig. 5a). There was no significantly high heterogeneity (I^2^ = 0%, *p* = 0.78).

### Asymptomatic ICH

The meta-analysis included 6 studies with 418 participants in the MT + angioplasty/stent group and 694 participants in the MT alone group. The pooled estimate showed no significant association of angioplasty/stenting and asymptomatic ICH (RR = 1.17, 95% CI = 0.86–1.60, *p* = 0.33) (Fig. 5b). There was no significantly high heterogeneity (I^2^ = 0%, *p* = 0.95).

### Any ICH

The meta-analysis included 7 studies with 544 participants in the MT + angioplasty/stent group and 861 participants in the MT alone group. The pooled estimate showed no significant association of angioplasty/stenting and any ICH (RR = 0.97, 95% CI = 0.77–1.21, *p* = 0.79) (Fig. 5c). There was no significantly high heterogeneity (I^2^ = 0%, *p* = 0.44).

### Mortality at 90 days

**Fig. 6:**
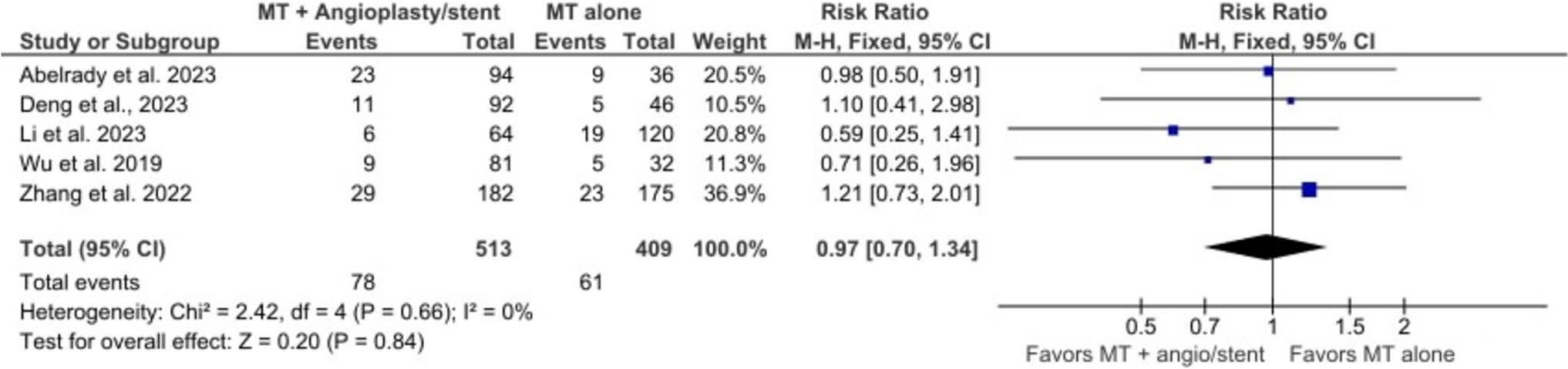
Forest plot shows mortality rate at 90 days of follow up

The meta-analysis included 5 studies with 513 participants in the MT + angioplasty/stent group and 409 participants in the MT alone group. The pooled estimate showed no significant association of angioplasty/stenting and 90-day mortality (RR = 0.97, 95% CI = 0.70–1.34, *p* = 0.84) (Fig. 6). There was no significantly high heterogeneity (I^2^ = 0%, *p* = 0.66).

### Subgroup analysis

Subgroup analysis based on the degree of arterial stenosis (> 70% stenosis) didn’t reveal any significant change from the main analysis. Angioplasty and/or stent was significantly better in terms of functional independence at 90 days (mRS = 0–2) and early neurological deterioration, while no significant difference was found compared to MT alone in terms of vessel recanalization symptomatic ICH, asymptomatic ICH, any ICH, and 90-day mortality. (Figure S1). Subgroup analysis based on the type of intervention (stent with or without angioplasty) revealed that stent with or without angioplasty was significantly better in terms of functional independence at 90 days (mRS = 0–2), while no significant difference was found compared to MT alone in terms of vessel recanalization, early neurological deterioration, asymptomatic ICH, and any ICH (Figure S2).

### Publication bias

The funnel plots, due to small number of studies, were not conclusive (Figure S3 to S10). Egger tests showed insignificant bias for vessel recanalization (*p* = 0.11), functional status at 90 days with mRS 0–2 (*p* = 0.37), early neurological deterioration (*p* = 0.73), symptomatic ICH (*p* = 0.13), asymmetric ICH (*p* = 0.40), any ICH (*p* = 0.61), and 90-day mortality (*p* = 0.19). Egger tests for functional status at 90 days with mRS 0–1 could not be estimated due to only including two studies. It also should be noted that in all of the outcomes, the Egger test included less than ten studies, which make this test less accurate and the findings should be interpreted cautiously.

## Discussion

ICAS-LVO greatly increases the risk of both stroke recurrence and poorer prognosis even following successful MT and appropriate medical therapy [11, 23]. Therefore, it is reasonable to hypothesize that correcting the underlying ICAS that caused the LVO using MT + angioplasty/stenting would lead to improved outcomes. This systematic review and meta-analysis sought to determine the efficacy and safety of mechanical thrombectomy + angioplasty/stenting versus mechanical thrombectomy alone in patients with ICAS-LVO.

One primary outcome of this study was vessel recanalization. This is an important marker since nearly half of ICAS-LVO may require rescue angioplasty and/or stenting due to reocclusion [23]. An anticipated advantage of MT + angioplasty/stent would be a greater occurrence of vessel recanalization without subsequent reocclusion because it directly addresses the underlying ICAS. While this meta-analysis found no statistically significant differences (as an overall effect or for individual studies) in vessel recanalization, MT + angioplasty/stent was slightly favored compared to MT alone. The marginal difference between MT + angioplasty/stent and MT alone did not appear to be influenced by the patient population, nor by the affected vessel. Abelrady et al. 2023 and Wu et al. 2019 most clearly favored MT + angioplasty/stent [1, 25]. These studies occurred in geographically distinct areas with vastly different patient populations. This is an important factor given the well-established geographical and racial determinants of ICAS [7, 15]. Furthermore, while Abelrady et al. 2023 had a significant number of patients with posterior circulation LVOs (37%), Wu et al. 2019 included only patients with LVOs of the anterior circulation [1, 25]. Given the disparities in these studies, it is promising that vessel recanalization was more common in the MT + angioplasty/stent arm compared to MT alone, despite not reaching statistical significance. There is likely a need to procure a larger patient cohort to establish the ultimate impact MT + angioplasty/stent may have on vessel recanalization.

Functional independence at 90 days for mRS = 0–2 and mRS = 0–1 were the other primary outcomes of this study. Functional independence at 90 days for mRS = 0–2 had promising results. Patients receiving MT + angioplasty/stent were 24% more likely to achieve functional independence at 90 days (mRS = 0–2). Functional independence at 90 days (mRS = 0–1) was also more likely in the MT + angioplasty/stent; however, this result was not statistically significant. The most likely cause of the statistical discrepancy when evaluating mRS = 0–2 compared to mRS = 0–1 was the number of applicable studies (9 versus 2, respectively). The improved functional outcomes may be directly associated with the lower propensity for patients who underwent MT + angioplasty/stent to develop reocclusion. It has been previously established that nearly half of the recanalized arteries in ICAS-LVO will reocclude within 3 days [7]. While subsequent reocclusion was not assessed in this study, it is reasonable to posit that additional angioplasty and/or stent lowered both the short- and long-term likelihood of developing reocclusion. The increased blood flow resulting from the correction of the underlying ICAS can contribute to angiogenesis [5]. Angiogenesis is a major contributor to healing following ischemic stroke [9]. Furthermore, the time to intervention was not an included component of the studies included in the meta-analysis. Patients receiving MT + angioplasty/stent would be expected to further benefit from the increased blood flow if it is established within a few hours of symptom onset [21]. Overall, while the mechanism remains unknown, MT + angioplasty/stent seemed to provide better functional outcomes compared to MT alone. However, to confirm the results and possibly explain the discrepancy findings in terms of mRS = 0–2 and mRS = 0–1, a larger sample size is necessary.

The addition of angioplasty and/or stent comes with risks. Common adverse events include hemorrhage, infection, and dissection. Stroke, being the inciting event leading to the intervention, and intervention to the middle cerebral artery (MCA) are independently associated with increased risks of experiencing these adverse events [19, 26]. The MCA was the most common site of LVO for all studies included in this meta-analysis. Therefore, both risk factors (stroke as the inciting event and MCA location) are very pertinent in determining the safety profile of MT + angioplasty/stent. Promisingly, despite the additional intervention, the MT + angioplasty/stent arm did not have a significantly increased risk of adverse events. However, the MT + angioplasty/stent arm had a mildly increased risk for symptomatic and asymptomatic ICH. This is expected given that hemorrhage is a common adverse event from angioplasty and/or stent procedures [6]. Furthermore, distal vessel damage due to ischemia and inflammation occurs following an acute ischemic event. Therefore, increased perfusion pressure in this setting may predispose individuals to an ischemic to hemorrhagic transformation. Promisingly, patients in the MT + angioplasty/stent group had a lower risk of experiencing any ICH, which likely reflects that angioplasty and/or stent did not make an appreciable difference in causing hemorrhage, as shown by the lack of statistical significance. In addition; mortality had decreased rates in the MT + angioplasty/stent arm, but didn’t reach statistical significance much like the other findings of adverse events; however, given that this group experienced an additional procedure, it is an encouraging sign that MT + angioplasty/stent is a safe intervention. Furthermore, the MT + angioplasty/stent group had a significantly lower incidence of early neurological deterioration, which is mostly caused by ICH, reocclusion, and infarct expansion [16], showing that angioplasty/stent may play a protective function in ICH, reocclusion, and infarct expansion. However, larger sample size is still needed before draw firm conclusions.

### Limitations

The primary limitation of this study is the lack of randomized controlled trials. Given the debate that surrounds the use of MT + angioplasty/stent for ICAS-LVO, a conclusive clinical trial would be necessary to establish MT + angioplasty/stent as the gold standard intervention. It should be noted that the small sample size included in this meta-analysis may have limited the statistical impact that MT + angioplasty/stent has on rarer events. While publication bias or risk of bias assessment did not appear to be an issue, the population characteristics of the included studies have obvious implications. This is particularly true given the nature of ICAS having a greater prevalence in patients of certain racial groups and behavioral habits [13]. A future clinical trial would enable better control of confounding factors and effect modifiers, such as antiplatelet regimen, stent type, or timing of angioplasty/stenting.

## Conclusions

In patients with ICAS-LVO, MT + angioplasty/stent significantly increased functional independence at 90 days and decreased early neurological deterioration. MT + angioplasty/stent also led to a small but insignificant increase in recanalization rate. There was no statistically significant difference in adverse events between MT + angioplasty/stent versus MT alone, but there was variation in risk profile between the two approaches. MT + angioplasty/stent was at lower risk of any ICH, and mortality at 90 days. However, MT + angioplasty/stent increased the risk of symptomatic and asymptomatic ICH.

## Supplementary Information

Below is the link to the electronic supplementary material.

ESM 1 Supplementary Material 1 (DOCX 534 KB)

## Data Availability

No datasets were generated or analysed during the current study.
