# Supplementary material for: Angioplasty and/or stenting after thrombectomy in patients with large vessel occlusion associated with underlying intracranial atherosclerotic stenosis: a meta-analysis and systematic review
Source: Acta Neurochir (Wien). 2025 Oct 16;167(1):274. doi: 10.1007/s00701-025-06690-6 (PMC12531308; doi:10.1007/s00701-025-06690-6)
Supplement: Supplementary file 1 — Supplementary Material 1 (DOCX 534 KB) [file 701_2025_6690_MOESM1_ESM.docx]

**Figures legends**

**Figure S1:** Forest plots of subgroup analysis of patients with stenosis > 70% in terms of (a) vessel recanalization, (b) functional independence at 90-days (mRS 0-2), (c) early neurological deterioration, (d) symptomatic ICH, (e) asymptomatic ICH, (f) any ICH, and (g) 90-day mortality.

**Figure S2:**  Forest plots of subgroup analysis of patients who received stent with or without angioplasty in terms of (a) vessel recanalization, (b) functional independence at 90-days (mRS 0-2), (c) early neurological deterioration, (d) asymptomatic ICH, and (e) any ICH.

**Figure S3:** Funnel plot of vessel recanalization.

**Figure S4:** Funnel plot of functional independence at 90-days (mRS 0-2).

**Figure S5:** Funnel plot of functional independence at 90-days (mRS 0-1).

**Figure S6:** Funnel plot of early neurological deterioration.

**Figure S7:** Funnel plot of symptomatic ICH.

**Figure S8:** Funnel plot of asymptomatic ICH.

**Figure S9:** Funnel plot of any ICH.

**Figure S01:** Funnel plot of 90-day mortality.


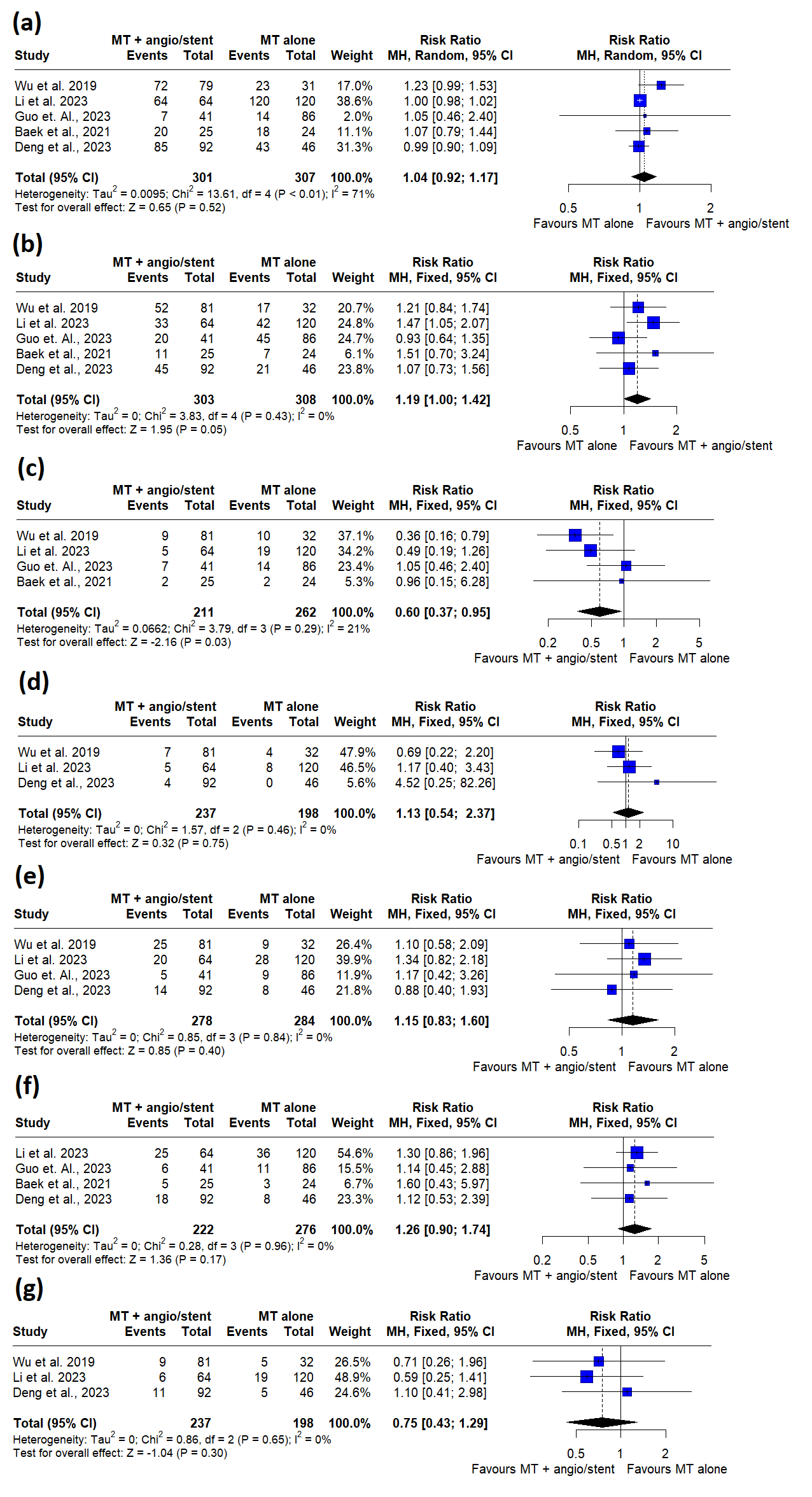


**Figure S1:** Forest plots of subgroup analysis of patients with stenosis > 70% in terms of (a) vessel recanalization, (b) functional independence at 90-days (mRS 0-2), (c) early neurological deterioration, (d) symptomatic ICH, (e) asymptomatic ICH, (f) any ICH, and (g) 90-day mortality.


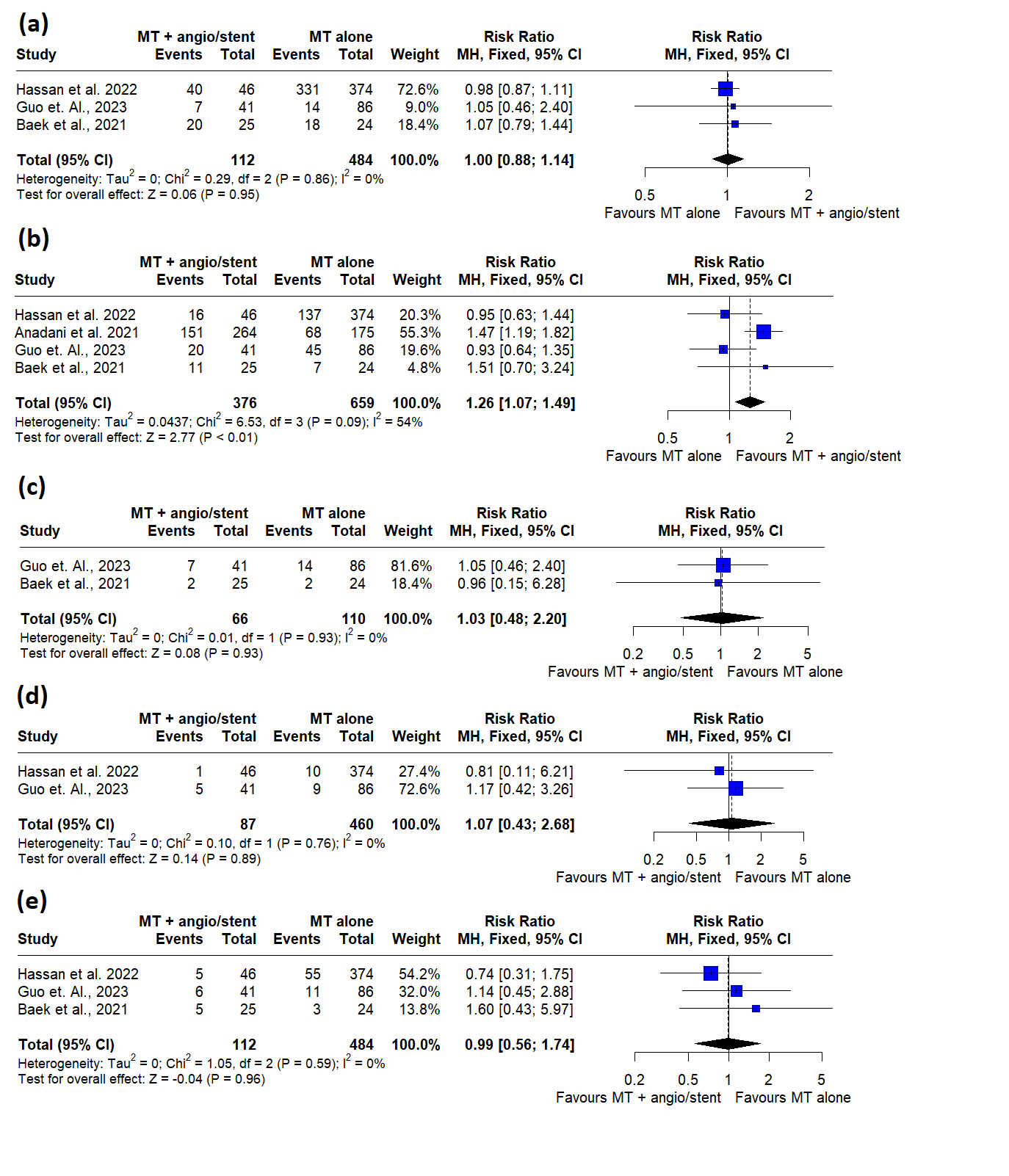


**Figure S2:**  Forest plots of subgroup analysis of patients who received stent with or without angioplasty in terms of (a) vessel recanalization, (b) functional independence at 90-days (mRS 0-2), (c) early neurological deterioration, (d) asymptomatic ICH, and (e) any ICH.


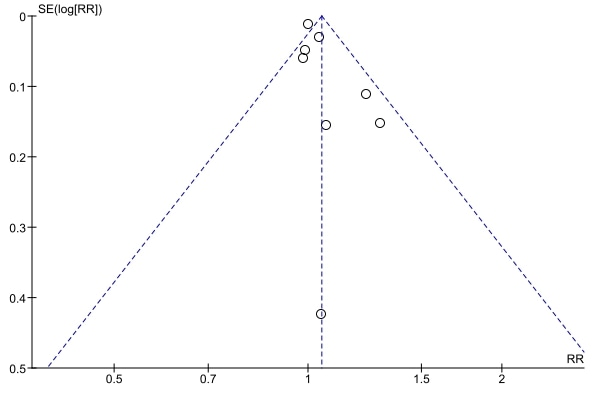


**Figure S3:** Funnel plot of vessel recanalization.


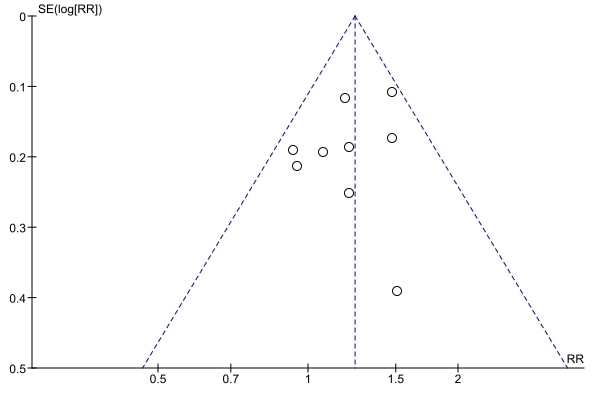


**Figure S4:** Funnel plot of functional independence at 90-days (mRS 0-2).


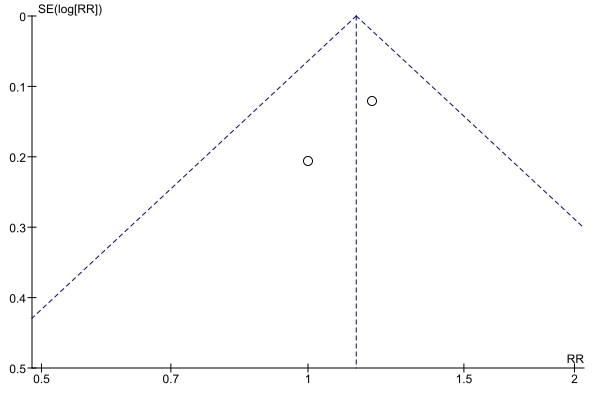


**Figure S5:** Funnel plot of functional independence at 90-days (mRS 0-1).


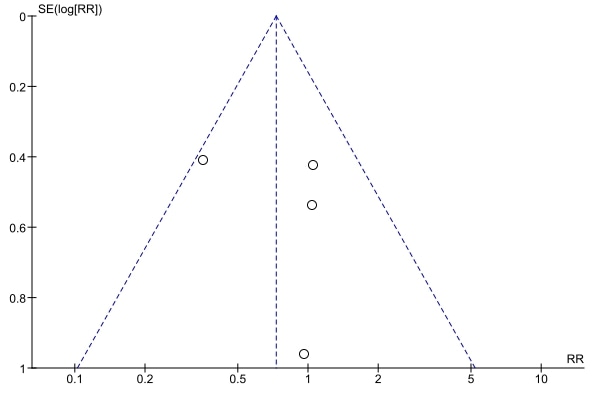


**Figure S6:** Funnel plot of early neurological deterioration.


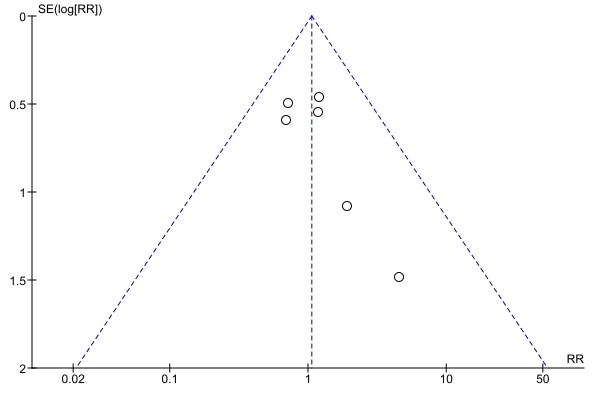


**Figure S7:** Funnel plot of symptomatic ICH.


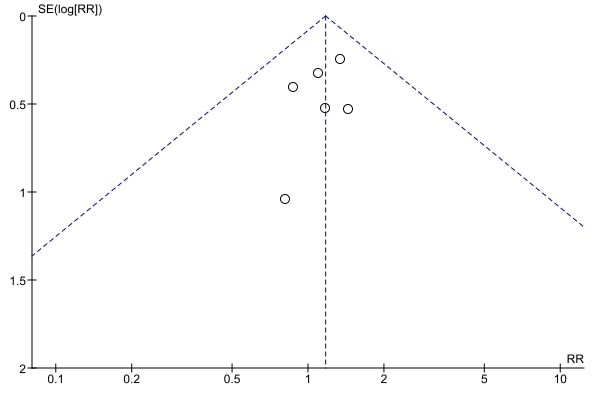


**Figure S8:** Funnel plot of asymptomatic ICH.


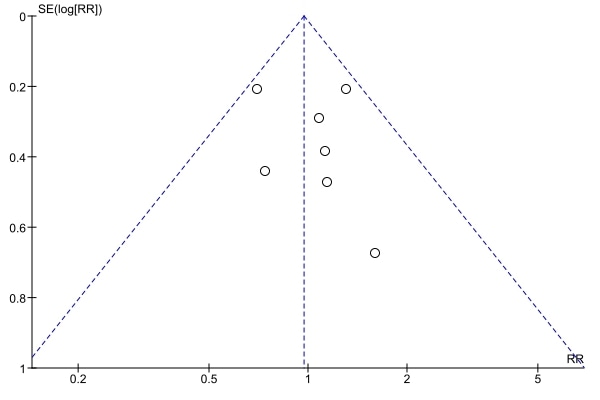


**Figure S9:** Funnel plot of any ICH.


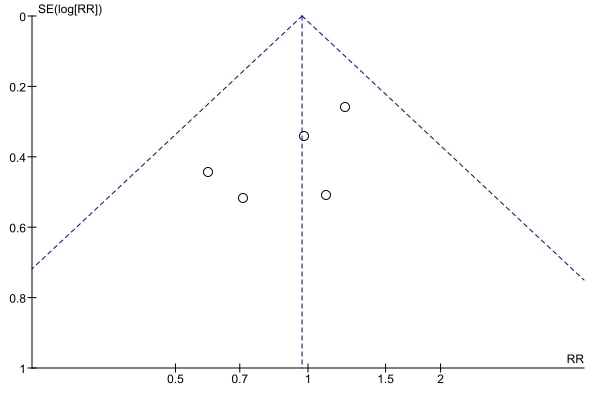


**Figure S10:** Funnel plot of 90-day mortality.
